# Physical Activity and Liver Fibrosis: A Stratified Analysis by Obesity and Diabetes Status

**DOI:** 10.3390/jcm15020757

**Published:** 2026-01-16

**Authors:** Junghwan Cho, Sunghwan Suh, Ji Min Han, Hye In Kim, Hanaro Park, Hye Rang Bak, Ji Cheol Bae

**Affiliations:** 1 Division of Endocrinology and Metabolism, Department of Internal Medicine, Samsung Changwon Hospital, Sungkyunkwan University School of Medicine, Changwon 51353, Republic of Korea; whwjdzl1004@gmail.com (J.C.); sh23.suh@samsung.com (S.S.); jimin1981@naver.com (J.M.H.); hyein11.kim@gmail.com (H.I.K.); 2 Department of Otorhinolaryngology-Head & Neck Surgery, Samsung Changwon Hospital, Sungkyunkwan University School of Medicine, Changwon 51353, Republic of Korea; naronaro1983@gmail.com; 3 Department of Family Medicine, Samsung Changwon Hospital, Sungkyunkwan University School of Medicine, Changwon 51353, Republic of Korea; hyerangbak@gmail.com

**Keywords:** exercise, liver fibrosis, diabetes mellitus, type 2

## Abstract

**Background/Objectives**: We investigated the association between leisure-time physical activity (LTPA) and liver fibrosis, and whether this relationship differs by obesity and diabetes status. **Methods**: We conducted a cross-sectional analysis using data from the National Health and Nutrition Examination Survey (NHANES) 2017–March 2020 cycle. LTPA was assessed using the Global Physical Activity Questionnaire (GPAQ) and classified as physically active if engaging in ≥600 metabolic equivalent (MET)-minutes per week of moderate-to-vigorous activity, or inactive. Clinically significant liver fibrosis was defined as liver stiffness measurement (LSM) ≥ 8.0 kPa on transient elastography. Multivariable logistic and linear regression models estimated adjusted odds ratios (ORs) for significant liver fibrosis, with additional subgroup analyses according to obesity and diabetes status. **Results**: In 7662 U.S. adults, physically active participants (n = 2721) had a lower prevalence of significant fibrosis than inactive individuals (5.4% vs. 11.4%, *p* < 0.001). In multivariable analysis, Participants who were physically active were associated with 42% lower odds of having fibrosis (OR 0.58, 95% confidence interval [CI] 0.41–0.82; *p* = 0.004). This association remained consistent in subgroup analyses stratified by obesity and diabetes status, even in the non-obese subgroup with body mass index (BMI) < 30 kg/m^2^ (OR 0.54, 95% CI 0.32–0.91; *p* = 0.022) and the non-diabetic subgroup (OR 0.59, 95% CI 0.39–0.90; *p* = 0.016). **Conclusions**: Regular moderate-to-vigorous LTPA was independently associated with lower likelihood of clinically significant liver fibrosis. This beneficial association was significant regardless of obesity or diabetes status, suggesting that LTPA may play a clinically meaningful role in populations at high risk for progressive liver disease.

## 1. Introduction

Metabolic dysfunction-associated steatotic liver disease (MASLD), formerly known as non-alcoholic fatty liver disease, is now recognized as one of the leading causes of chronic liver disease worldwide [1]. Within its spectrum, liver fibrosis is a key factor associated with progression to advanced liver disease, including cirrhosis, hepatocellular carcinoma, and liver-related mortality [2]. Fibrosis progression primarily occurs in patients with metabolic dysfunction-associated steatohepatitis (MASH), as steatohepatitis is the main cause of fibrosis progression in MASLD [3]. Among patients with MASLD, those with type 2 diabetes (T2D) or obesity are at a substantially higher risk of developing MASH [4]. Therefore, current guidelines, including those from American Diabetes Association, the European Association for the Study of Diabetes, and the European Association for the Study of the Liver, recommend screening for liver fibrosis in adults with T2D or obesity using a calculated fibrosis-4 index and liver stiffness measurement (LSM) via transient elastography [4,5].

Insulin resistance is a pivotal pathophysiological feature of various metabolic disorders, playing both causal and consequential roles in conditions such as obesity, T2D, and MASLD [6]. Its presence increases the risk of metabolic dysfunction, making it a critical therapeutic target in the management of metabolic disease [7]. Physical activity contributes to both the prevention and improvement of insulin resistance through immediate and long-term mechanisms [8]. These effects are observed in both healthy individuals and those with metabolic disorders, even regardless of weight reduction [9]. In the context of MASLD, numerous clinical studies have shown that physical activity confers beneficial effects, particularly through significant reductions in hepatic steatosis [10,11]. However, many of these studies have been limited by small sample sizes, making it difficult to assess generalizability [12,13,14].

Therefore, we aimed to investigate the association between physical activity and liver fibrosis, and to examine whether this association differs according to obesity and diabetes status, using a nationally representative sample of United States (U.S.) adults. We hypothesized that being physically active would be associated with a lower likelihood of significant liver fibrosis, with potential variation in this association by obesity and diabetes status.

## 2. Materials and Methods

### 2.1. Data Source and Subjects

We conducted a cross-sectional analysis using data from the National Health and Nutrition Examination Survey (NHANES) collected from 2017 through March 2020, prior to the COVID-19 pandemic. Data collected from 2019 to March 2020 were combined with data from the NHANES 2017–2018 cycle. The NHANES, conducted by the National Center for Health Statistics (NCHS), includes a nationally representative sample of the U.S. population across all age groups. Detailed information on the NHANES design and methodology is available elsewhere [15]. In brief, participants underwent a structured interview and health exams. The interviews included questions on demographics, socioeconomic status, dietary behaviors, physical activity, and health. The examinations consisted of medical and physical measurements, as well as laboratory tests, all performed by highly trained medical personnel. In addition, liver transient elastography was performed on participants aged 12 years and older. The NHANES protocol (Protocols #2011-17 and #2018-01) was approved by the NCHS Research Ethics Review Board, and written informed consent was obtained from all participants.

**Figure 1:**
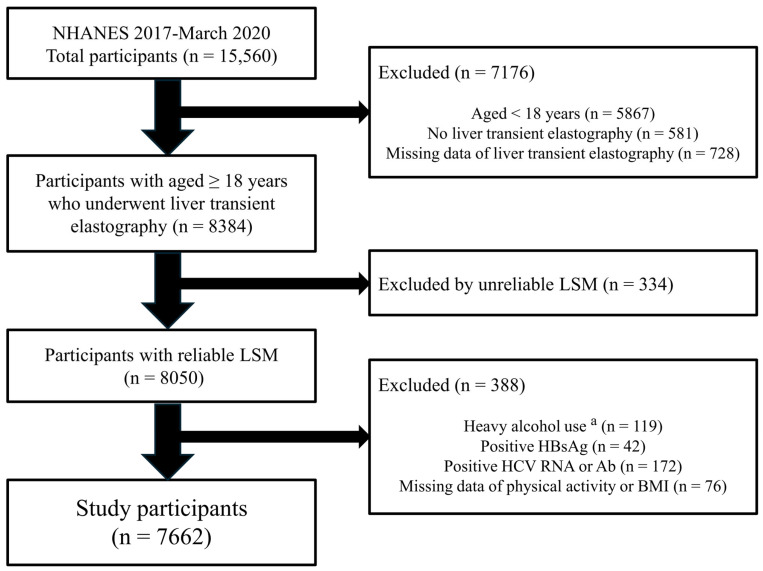
The selection process for eligible study participants. ^a^ Weekly alcohol intake >420 g for male and >350 g for female. LSM, liver stiffness measurement; HBsAg, hepatitis B surface antigen; HCV RNA, hepatitis C viral RNA; Ab, antibody; BMI, body mass index.

Figure 1 shows the selection process for eligible study participants. Among the total participants (n = 15,560) in the NHANES 2017–March 2020 Pre-Pandemic cycle, 7176 subjects were excluded: aged < 18 years (n = 5867), not undergoing liver transient elastography (n = 581), or missing results of elastography (n = 728). Of those aged ≥ 18 years who underwent liver transient elastography, 334 were further excluded by unreliable LSM. Liver stiffness evaluation was considered unreliable if fewer than 10 valid measurements were obtained, or if the ratio of the interquartile range (IQR) to the median LSM (IQR/M) exceeded 0.30. Participants with reliable LSM were further excluded based on the following criteria: (1) heavy alcohol use, weekly intake > 420 g for male and > 350 g for female (n = 119); (2) positive hepatitis B surface antigen (n = 42); (3) positive hepatitis C viral RNA or antibody (n = 172); (4) missing data of body mass index (BMI) or physical activity (n = 76). After applying these exclusion criteria, a total of 7662 participants were included in the final analysis.

### 2.2. Physical Activity

We utilized the NHANES 2017–March 2020 Pre-pandemic physical activity questionnaire data [15]. Physical activity was assessed based on participants’ responses to the Global Physical Activity Questionnaire (GPAQ), which was administered by trained interviewers. Only leisure-time physical activity (LTPA) domains of the GPAQ were used for the present analysis. Occupational, household, and transportation-related activities were not included in the present analysis. Participants were asked to report the number of days per typical week they engaged in vigorous- and moderate- intensity sports, fitness, and recreational activities, and the average amount of time spent per day on these activities. Participants were classified as physically active if they met the World Health Organization (WHO) recommendations of at least 150 min per week of moderate-intensity activity, 75 min per week of vigorous-intensity activity, or an equivalent combination, corresponding to a total of ≥600 metabolic equivalent (MET)-minutes per week [16,17]. MET-minutes were calculated by multiplying the reported frequency (days per week), duration (minutes per day), and assigned MET values (4.0 METs for moderate-intensity and 8.0 METs for vigorous-intensity activities), based on the WHO GPAQ scoring protocol [16].

### 2.3. Liver Ultrasound Transient Elastography

Liver transient elastography was performed with the FibroScan 502 Touch (Echosens, Paris, France), using either a medium or extra-large probe. The XL probe was used when the distance between the skin and liver capsule was ≥25 mm, which accounted for approximately 26% of all participants. Liver stiffness was determined by assessing the propagation speed of a 50 Hz shear wave through hepatic tissue. The measured velocity is converted into liver stiffness, expressed in kilopascals (kPa), where higher values indicate greater stiffness. Hepatic steatosis was quantified by controlled attenuation parameter (CAP), expressed in decibels per meter (dB/m). Both LSM and CAP provided by FibroScan are established noninvasive markers for assessing hepatic fibrosis and steatosis, respectively. Participants were asked to fast for a minimum of three hours before undergoing the examination. Multiple LSM and CAP values were obtained from the right hepatic lobe through the intercostal space at the intersection of the mid-axillary line and a transverse plane at the level of the xiphoid process, with the participant in the supine position. The median of valid measurements was recorded as the final value for both LSM and CAP. All elastography assessments were conducted by NHANES health technicians who had received standardized training and certification under supervision by NHANES experts, following the protocol recommended by the device manufacturer.

### 2.4. Definition and Measurements

In this study, LSM threshold of 8.0 kPa was used to define clinically significant liver fibrosis, corresponding to METAVIR histological fibrosis stage ≥ F2, and obesity was defined as a BMI of ≥30 kg/m^2^ [4]. Participants were classified as having diabetes if they had a fasting plasma glucose level of ≥126 mg/dL, glycated hemoglobin (HbA1c) level of ≥ 6.5%, or reported current use of glucose-lowering medications [18]. Trained health technicians collected the body measurements. BMI was calculated by dividing weight in kilograms by height in meters squared, and the result was rounded to one decimal point. Smokers were defined as smoking at least 100 cigarettes in their lifetime, a key threshold used in health surveys. Alcohol use was defined as above MASLD limit of weekly intake > 210 g for males and >140 g for females. Household income was assessed using the poverty–income ratio (PIR), defined as the ratio of family income to the federal poverty threshold according to household size and survey year. For analysis, PIR was categorized using a cutoff of 1.3 to indicate low household income. Education level was categorized into less than high school, and high school or above. Data on demographic characteristics and medical history was obtained in household interviews by trained interviewers using a computer-assisted personal interviewing system. Participants were instructed to fast for 9 h prior to blood sample collection. Details of measurements are available on the NHANES website provided by the Centers for Disease Control and Prevention [15].

### 2.5. Statistical Analysis

The clinical characteristics were compared between physically active and inactive participants using survey-weighted linear regression for continuous variables and survey-weighted (Rao-Scott) chi-square tests for categorical variables, accounting for the NHANES complex sampling design. Survey-weighted logistic regression models were used to estimate the odds ratios (ORs) for clinically significant liver fibrosis (LSM ≥ 8.0 kPa) according to physical activity status. We further conducted subgroup analysis to examine whether the association between physical activity status and liver fibrosis differed by diabetes and obesity status, with interaction term included in the survey-weighted logistic regression models to test for effect modification. Adjusted LSM values and predicted probabilities of significant liver fibrosis across the range of BMI values were estimated using the margins command in Stata, based on survey-weighted multivariable linear and logistic regression models, respectively, that included a BMI-physical activity interaction term. In participants stratified by diabetes status, difference in LSM values between physically active and inactive groups were assessed using survey-weighted analysis of covariance (ANCOVA) implemented via linear regression models, adjusting for potential confounders.

Missing data were handled using variable-specific approaches. Participants with missing BMI, physical activity, or liver transient elastography measurements were excluded from the analytic cohort. For laboratory variables (fasting glucose, HbA1c, aspartate aminotransferase [AST]; alanine aminotransferase [ALT], triglycerides, and high-density lipoprotein cholesterol [HDL-C]), estimates were calculated among participants with available measurements, with missingness ranging from approximately 345 to 556 participants. Alcohol consumption had missing values (n = 395); therefore, percentage in descriptive analyses were calculated excluding missing data, whereas a missing-indicator approach was used in regression models. Missing values for PIR and education level were imputed using multiple imputation, and imputed values were incorporated into all survey-weighted estimates and regression models. All analyses were performed using Stata program version 15.1 (Stata Corp., College Station, TX, USA).

## 3. Results

### 3.1. Cohort Characteristics

In this cross-sectional study of 7662 U.S. adults, the mean age was 46.9 ± 0.6 years, 44.7% were men, and the average BMI was 29.4 ± 0.17 kg/m^2^. The overall prevalence of diabetes was 12.4%, 40.1% of participants were classified as obese (BMI ≥ 30 kg/m^2^), and 8.9% had clinically significant liver fibrosis (≥F2), defined as LSM ≥ 8.0 kPa (Table 1). The prevalence of clinically significant fibrosis was substantially higher among obese participants (16.3%) compared with non-obese individuals (4.0%). Clinically significant fibrosis was observed in 25.9% of individuals with diabetes, whereas the prevalence was 6.5% among those without diabetes.

### 3.2. Clinical Characteristics by Leisure-Time Physical Activity

The clinical characteristics of participants by physical activity are shown in Table 1. Among the study population, 2721 (35.5%) were categorized as physically active. Compared to the inactive group, physically active individuals were significantly younger and more likely to be male, had lower smoking rates, higher household income and education levels. They also had a lower mean BMI and a lower prevalence of obesity (31.8% vs. 45.9%, *p* < 0.001), as well as reduced HbA1c levels and a lower prevalence of diabetes (7.8% vs. 15.6%, *p* < 0.001). Although there were no significant differences in ALT levels between groups, hepatic steatosis, as measured by the CAP, was significantly lower in the physically active group (249.2 ± 2.3 vs. 270.9 ± 1.1 dB/m, *p* < 0.001). Notably, LSM was also lower in physically active individuals (5.2 ± 0.1 vs. 5.9 ± 0.1 kPa, *p* < 0.001), and the prevalence of clinically significant liver fibrosis (LSM ≥ 8.0 kPa) was nearly half in the physically active group compared to the inactive group (5.4% vs. 11.4%, *p* < 0.001). There were no significant differences in the distribution of race/ethnicity between the two groups.

### 3.3. Association Between Physical Activity and Liver Fibrosis

Table 2 shows the association between physical activity and clinically significant liver fibrosis. After adjustment for age, sex, BMI, and presence of diabetes mellitus, physically active participants had 45% lower odds of having significant fibrosis compared to inactive participants (OR 0.55, 95% confidence interval [CI] 0.39–0.77; *p* 0.001). After additional adjustment for alcohol use, cigarette smoking, race/ethnicity, education, and household income, the association remained significant (OR 0.58, 95% CI 0.41–0.82; *p* 0.004). In a sensitivity analysis performed using an alternative threshold of LSM ≥ 7.5 kPa, this association remained consistent with the primary analysis (Supplementary Table S1). A subgroup analysis examined whether this inverse association between physical activity and fibrosis differed by obesity and diabetes status (Table 3). Regardless of the presence (BMI ≥ 30 kg/m^2^) or absence (BMI < 30 kg/m^2^) of obesity, physically active individuals had substantially lower odds of having significant fibrosis compared with inactive participants (*p* for interaction = 0.736). Similarly, participants who were physically active had a significantly lower odds of having significant fibrosis, regardless of whether they had diabetes (*p* for interaction = 0.494). The sensitivity analysis results for the subgroups also confirmed a consistent association, similar to the primary analysis (Supplementary Table S2).

**Figure 2:**
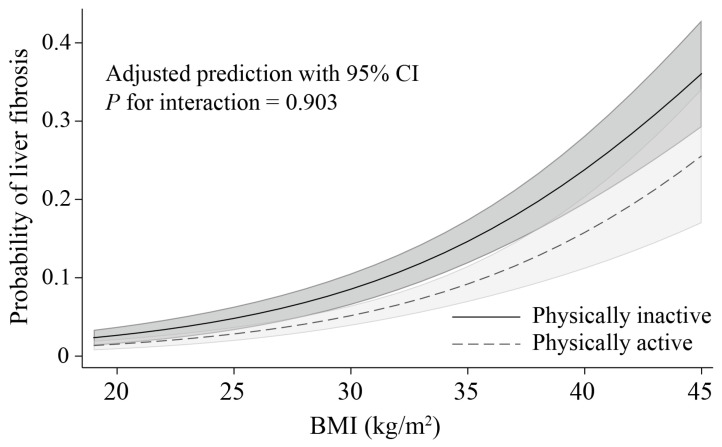
Adjusted probability of clinically significant liver fibrosis across the range of BMI by physical activity status (n = 7662). Values are shown as predicted probabilities with 95% confidence intervals. Models were adjusted for age, sex, presence of diabetes, alcohol use, cigarette smoking, race/ethnicity, education, and household income. BMI, body mass index; CI, confidence interval.

Figure 2 shows the adjusted predicted probability of clinically significant liver fibrosis across the range of BMI according to physical activity status. In both physically active and inactive participants, the probability of fibrosis increased progressively with increasing BMI. Physically active individuals consistently had lower predicted probabilities of fibrosis compared with physically inactive individuals, across the entire BMI range. There was no significant interaction between BMI and physical activity (*p* for interaction = 0.903), indicating that the inverse association between physical activity and fibrosis was maintained across BMI levels.

**Figure 3:**
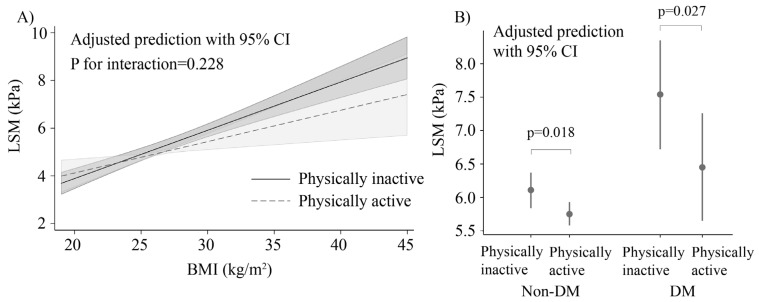
Adjusted liver stiffness measurement (LSM) according to BMI, physical activity, and diabetes status. (**A**) Predicted mean LSM across the range of BMI by physical activity status in the overall population. (**B**) Adjusted mean LSM by physical activity stratified by diabetes status. Models were adjusted for age, sex, diabetes status (Panel (**A**) only), obesity status (Panel (**B**) only), alcohol use, cigarette smoking, race/ethnicity, education, and household income. BMI, body mass index; CI, confidence interval.

A similar association was observed for LSM, which increased progressively with BMI. In survey-weighted linear regression models, liver stiffness increased progressively with higher BMI in both physically active and inactive participants (Figure 3A). Physically active individuals tended to have lower adjusted LSM values than physically inactive individuals across a wide range of BMI. There was no significant interaction between BMI and physical activity (*p* for interaction = 0.228). When stratified by diabetes status, physically active participants had significantly lower adjusted LSM values than physically inactive participants in both non-diabetic (*p* 0.018) and diabetic groups (*p* 0.027) (Figure 3B).

## 4. Discussion

Results from the 2017 to 2020 NHANES survey show that 7662 of a national representative sample of U.S. adults had clinically significant liver fibrosis (≥F2), while 35.5% reported engaging in regular LTPA at a moderate intensity or higher. In this population, regular LTPA was independently associated with lower odds of having clinically significant liver fibrosis. Notably, this inverse association remained consistent even after adjusting for major risk factors for liver fibrosis, such as obesity, diabetes, and demographic variables. In subgroup analyses stratified by obesity and diabetes status, significant associations were identified regardless of whether obesity or diabetes presence.

Given its correlation with liver disease progression, fibrosis is a key determinant of clinical outcomes, including cirrhosis and hepatocellular carcinoma. The risk of these liver-related events begins to increase once fibrosis develops [2,19]. Accumulating evidence indicates that liver fibrosis is potentially reversible, whereas cirrhosis is generally considered irreversible [2,3,20]. For this reason, recent screening strategies for MASLD and MASH have increasingly focused on the detection of fibrosis in at-risk individuals, highlighting the clinical importance of interventions that can mitigate fibrosis or slow its progression [4,5]. At the same time, in patients without fibrosis, efforts should be directed toward reducing hepatic fat content, the primary driver of disease, in order to prevent the development of fibrosis [5,21,22]. Numerous studies have demonstrated that regular physical activity effectively reduces hepatic steatosis [13,23], supporting its potential role in preventing liver fibrosis, while our findings further suggest that physical activity may confer benefit with respect to liver fibrosis. In addition to differences in the prevalence of fibrosis, our study demonstrated that, even at the same degree of obesity, physically active individuals consistently exhibited lower liver stiffness across most BMI levels. Well-established pathophysiologic mechanisms and prior epidemiologic evidence lend biological plausibility to our findings, supporting the physical activity as effective lifestyle modification against fibrosis [24,25].

Prior to our study, studies using NHANES 2017–2020 data also confirmed a significant association between LTPA and the liver fibrosis [26,27,28]. However, previous studies did not consider obesity and diabetes, which are major risk factors for liver fibrosis [26], or did not conduct additional stratified analyses according to their presence or absence [27,28]. T2D and obesity are the metabolic diseases that most strongly impact the natural history of MASLD, including progression to liver fibrosis and cirrhosis [4]. In insulin-resistant states, chronic hyperinsulinemia and hyperglycemia increase oxidative stress, promote systemic inflammation, and induce hepatic stellate cell activation, all of which drive fibrosis [29,30,31] and may blunt the beneficial effects of physical activity. This hypothesis of reduced effect has also been observed in prior trials of lifestyle modification, including diet and physical activity, where improvements in fibrosis were reduced in patients with T2D or severe obesity (BMI > 35 kg/m^2^) [10,29]. We performed subgroup analysis stratified by obesity and diabetes status, and the results showed that the effects of LTPA were significant regardless of whether obesity or diabetes presence. This suggests that LTPA itself is effective in lowering odds of liver fibrosis. In addition, previous studies demonstrating significant improvements in fibrosis through lifestyle modification primarily targeted severely obese individuals [12,14]. However, our study results showed that the effect of LTPA was significant not only in obese but also in non-obese individuals. (BMI < 30 kg/m^2^). Individuals who engage in LTPA tend to have a lower probability of liver fibrosis than those who are physically inactive, across the entire BMI range of our study. The lack of a significant interaction suggests that this association is observed consistently across the BMI spectrum rather than being confined to specific BMI ranges.

Our study has several limitations that should be considered. Due to its cross-sectional design, our study cannot determine causality. For example, people with more advanced fibrosis or poorer health could have less physical activity. Our interpretations rely on biological plausibility and previously established mechanisms. Although current physical activity status was assessed, we lacked information on the duration of regular physical activity (i.e., how long participants had been consistently engaging in physical activity) or whether participants achieved concomitant weight loss, which may have influenced the observed associations. We did not evaluate other types of PA besides LTPA (occupational, transport-related, etc.). There was a possibility of misclassification due to other domains of physical activity that were not analyzed, and the effects of these physical activities could not be evaluated. However, previous studies suggest that LTPA may be particularly relevant to liver fibrosis [26,27,28]. We did not consider dietary factors, which are integral components of lifestyle modification and may interact with physical activity in affecting outcomes. In addition, we did not differentiate diabetes type, and individuals with type 1 and type 2 diabetes were collectively classified as having diabetes, as this distinction was not available in NHANES data. Finally, medication use among participants with diabetes was not analyzed. The use of agents such as glucagon-like peptide-1 receptor agonists or sodium-glucose cotransporter 2 inhibitors may affect hepatic steatosis and fibrosis. Nevertheless, a key strength of our study lies in its use of real-world, nationally representative data. Unlike tightly controlled trials, our analysis reflects heterogeneity in the duration of regular LTPA participation, adherence, and weight loss status. These features enhance the clinical implication of our findings for the management of MASLD.

## 5. Conclusions

In this nationally representative sample of U.S. adults, we found that LTPA was associated with a lower prevalence and lower adjusted odds of clinically significant liver fibrosis. Physically active individuals also exhibited lower liver stiffness across the BMI spectrum, and this pattern was observed irrespective of diabetes status. These associations remained evident after adjustment for major sociodemographic, behavioral, and metabolic factors, indicating that the relationship between LTPA and liver fibrosis could not be explained simply by differences in obesity, diabetes, or socioeconomic status. In addition, these findings indicate that engagement in LTPA is associated with a lower burden of liver fibrosis across diverse metabolic risk groups, including individuals with obesity and diabetes, emphasizing that physical activity may play a clinically meaningful role in populations at high risk for progressive liver disease.

## Figures and Tables

**Table 1:** Clinical characteristics of the study participants by physical activity.

	Physically Inactive n = 4941	Physically Active n = 2721	*p*-Value	Total (n = 7662)
Age (year)	49.5 ± 0.3	43.2 ± 0.7	<0.001	46.9 ± 0.6
Male	44.7	54.5	<0.001	48.8
BMI (kg/m^2^)	30.3 ± 0.2	28.1 ± 0.3	<0.001	29.4 ± 0.17
Obese (BMI ≥ 30 kg/m^2^)	45.9	31.8	<0.001	40.1
Fasting glucose (mg/dL) ^a^	100.8 ± 0.6	95.5 ± 0.6	<0.001	98.6 ± 0.46
HbA1c (%) ^a^	5.7 ± 0.01	5.5 ± 0.02	<0.001	5.6 ± 0.01
Diabetes	15.6	7.8	<0.001	12.4
Triglyceride (mg/dL) ^a^	145.9 ± 2.9	128.2 ± 2.8	<0.001	138.6 ± 2.7
HDL-C (mg/dL) ^a^	52.4 ± 0.4	55.3 ± 0.6	<0.001	53.6 ± 0.4
AST (IU/L) ^a^	21.1 ± 0.19	21.8 ± 0.24	0.025	21.4 ± 0.2
ALT (IU/L) ^a^	22.4 ± 0.35	22.2 ± 0.37	0.673	22.3 ± 0.3
CAP (dB/m)	270.9 ± 1.1	249.2 ± 2.3	<0.001	262.0 ± 1.4
LSM (kPa)	5.9 ± 0.1	5.2 ± 0.1	<0.001	5.7 ± 0.1
LSM ≥ 8.0 kPa	11.4	5.4	<0.001	8.9
**Smoking**				
≥100 cigarettes in life	43.8	34.9	<0.001	40.1
**Alcohol use**				
above MASLD limit ^b^	6.0	6.3	0.708	6.1
**Household income**				
Low (PIR ≤ 1.3)	23.6	14.3	<0.001	19.8
Middle or higher (PIR > 1.3)	76.4	85.7		80.2
**Education**				
Less than high school	13.9	5.7	<0.001	10.6
High school or above	86.1	94.3		89.4
**Race/ethnicity**				
White	61.1	64.1	0.310	62.3
Hispanic	17.3	15.6		16.6
Black	11.8	10.3		11.2
Asian	5.7	6.2		5.9
other	4.1	3.8		4.0

Values are presented as survey-weighted means ± standard errors or weighted percentages. Group comparisons were performed using survey-weighted linear regression for continuous variables and Rao–Scott adjusted chi-square tests for categorical variables. Missing values for PIR and education level were imputed using multiple imputation. Alcohol consumption was estimated excluding participants with missing data (n = 395). Physically active refers to engagement in leisure-time physical activity. ^a^ Estimated among participants with available laboratory data. ^b^ weekly alcohol intake > 210 g for males and >140 g for females. BMI, body mass index; HbA1c, glycated hemoglobin; AST, aspartate aminotransferase; ALT, alanine aminotransferase; CAP, controlled attenuation parameter; LSM, liver stiffness measurement; MASLD, Metabolic Dysfunction-Associated Steatotic Liver Disease; PIR, poverty income ratio.

**Table 2:** Odds ratio for significant liver fibrosis according to physical activity status.

Variables	Physically Inactive	Physically Active	*p*-Value
Subjects, n	4941	2721	
Fibrosis, n (%)	11.4	5.4	
Adjusted odds ratio for fibrosis (95% CI)			
Age and sex	1	0.45 (0.32–0.62)	<0.001
Age, sex, obesity status, and presence of diabetes	1	0.55 (0.39–0.77)	0.001
Multivariable ^a^	1	0.58 (0.41–0.82)	0.004

Percentages, odds ratios, and *p* values were all derived using survey-weighted analyses accounting for the NHANES complex sampling design. Physically active refers to engagement in leisure-time physical activity. ^a^ Adjusted for age, sex, obesity status, presence of diabetes, alcohol use, cigarette smoking, race/ethnicity, education level, and household income. CI, confidence interval; NHANES, National Health and Nutrition Examination Survey.

**Table 3:** Subgroup analysis of association between physical activity and liver fibrosis by obesity and diabetes status.

Subgroup	Physically Inactive (n = 4941)		Physically Active (n = 2721)		Odds Ratio (95% CI)			
	n	Fibrosis	n	Fibrosis	Inactive	Active	*p*-Value	*p* for Interaction
**Obesity**								0.736
BMI < 30 kg/m^2^ (n = 4539)	2728	5.2	1811	2.6	1	0.54 (0.32–0.91)	0.022 ^a^	
BMI ≥ 30 kg/m^2^ (n = 3123)	2213	18.7	910	11.4	1	0.57 (0.39–0.81)	0.003 ^a^	
**DM**								0.494
non-DM (n = 6389)	3955	8.1	2434	4.4	1	0.59 (0.39–0.90)	0.016 ^b^	
DM (n = 1273)	986	28.8	287	17.4	1	0.47 (0.25–0.78)	0.007 ^b^	

Percentages, odds ratios, and *p* values were all derived using survey-weighted analyses accounting for the NHANES complex sampling design. Physically active refers to engagement in leisure-time physical activity. ^a^ adjusted for age, sex, presence of diabetes, alcohol use, cigarette smoking, race/ethnicity, education, and household income; ^b^ adjusted for age, sex, obesity status, alcohol use, cigarette smoking, race/ethnicity, education, and household income. CI, confidence interval; BMI, body mass index; DM, diabetes mellitus.

## Data Availability

NHANES data are publicly available. For more information, please visit the official NHANES website: https://www.cdc.gov/nchs/nhanes/index.html (accessed on 27 November 2025).

## Supplementary Materials

The following supporting information can be downloaded at: https://www.mdpi.com/article/10.3390/jcm15020757/s1, Table S1: Odds ratio for significant liver fibrosis (LSM ≥ 7.5 kPa) according to physical activity status, Table S2: Subgroup analysis of association between physical activity and liver fibrosis (LSM ≥ 7.5 kPa) by diabetes and obesity status.

## Abbreviations

The following abbreviations are used in this manuscript:

LTPA	leisure-time physical activity
NHANES	National Health and Nutrition Examination Survey
GPAQ	Global Physical Activity Questionnaire
MET	metabolic equivalent
LSM	liver stiffness measurements
ORs, CI	odds ratios, confidence interval
BMI	body mass index
MASLD	metabolic dysfunction-associated steatotic liver disease
MASH	metabolic dysfunction-associated steatohepatitis
T2D	type 2 diabetes
U.S.	United States
NCHS	National Center for Health Statistics
IQR	interquartile range
WHO	World Health Organization
CAP	controlled attenuation parameter
HbA1c	glycated hemoglobin
PIR	poverty–income ratio
AST	aspartate aminotransferase
ALT	alanine aminotransferase
HDL-C	high-density lipoprotein cholesterol
