# Supplementary material for: Physical Activity and Liver Fibrosis: A Stratified Analysis by Obesity and Diabetes Status
Source: J Clin Med. 2026 Jan 16;15(2):757. doi: 10.3390/jcm15020757 (PMC12842459; doi:10.3390/jcm15020757)
Supplement: Supplementary file 1 [file jcm-15-00757-s001.zip › jcm-4040982-supplementary.pdf]

Supplementary Table S1. Odds ratio for significant liver fibrosis (LSM ≥ 7.5 kPa) according to physical activity status

**Variables**	**Physically inactive**	**Physically active**	***p*-Value**
Subjects, n	4941	2721	
Fibrosis, LSM ≥ 7.5 kPa (%)	13.9	7.1	
Adjusted odds ratio for fibrosis (95% CI)			
Age and sex	1	0.45 (0.32–0.62)	<0.001
Age, sex, obesity status, and presence of diabetes	1	0.59 (0.45–0.78)	0.001
Multivariable [a]	1	0.63 (0.48–0.83)	0.002

Percentages, odds ratios, and *p* values were all derived using survey-weighted analyses accounting for the NHANES complex sampling design. Physically active refers to engagement in leisure-time physical activity. [a] Adjusted for age, sex, obesity status, presence of diabetes, alcohol use, cigarette smoking, race/ethnicity, education level, and household income. CI, confidence interval; NHANES, National Health and Nutrition Examination Survey.

Supplementary Table S2. Subgroup analysis of association between physical activity and liver fibrosis (LSM ≥ 7.5 kPa) by diabetes and obesity status.

Subgroup	Physically inactive (n = 4941)		Physically active (n = 2721)		Odds ratio (95% CI)			
	n	Fibrosis, LSM ≥ 7.5 kPa (%)	n	Fibrosis, LSM ≥ 7.5 kPa (%)	Inactive	Active	*p*-Value	*p* for Interaction
**Obesity**								0.660
BMI < 30 kg/m^2 (n=4539)	2728	6.3	1811	3.8	1	0.60 (0.39–0.94)	0.028 [a]	
BMI ≥ 30 kg/m^2 (n=3123)	2213	22.8	910	14.1	1	0.59 (0.42–0.82)	0.003 [a]	
**DM**								0.482
non-DM (n=6389)	3955	10.3	2434	5.9	1	0.67 (0.49–0.91)	0.014 [b]	
DM (n=1273)	986	33.2	287	21.1	1	0.49 (0.28–0.85)	0.012 [b]	

Percentages, odds ratios, and p values were all derived using survey-weighted analyses accounting for the NHANES complex sampling design. Physically active refers to engagement in leisure-time physical activity. [a] adjusted for age, sex, presence of diabetes, alcohol use, cigarette smoking, race/ethnicity, education, and household income; [b] adjusted for age, sex, obesity status, alcohol use, cigarette smoking, race/ethnicity, education, and household income. CI, confidence interval; BMI, body mass index; DM, diabetes mellitus.
